# The positive association between white blood cell count and metabolic syndrome is independent of insulin resistance among a Chinese population: a cross-sectional study

**DOI:** 10.3389/fimmu.2023.1104180

**Published:** 2023-04-28

**Authors:** ZhongYu Ren, Shi Luo, Lian Liu

**Affiliations:** ^1^ School of Physical Education, Southwest University, Chongqing, China; ^2^ School of Physical Education, Huzhou University, Huzhou, China

**Keywords:** cross-sectional study, insulin resistance, white cell counts, metabolic syndrome, Chinese population

## Abstract

**Background:**

The association between white blood cells (WBCs) and metabolic syndrome (MS) has been consistently reported in previous studies using regional samples. However, it remains unclear whether this relationship has urban–rural differences and is independent of insulin resistance using a large-scale representative sample. Additionally, accurate risk prediction in patients with MS is crucial for developing targeted interventions to enhance the quality of life and prognosis of patients.

**Aims:**

The aims of this study were (1) to examine the cross-sectional association between WBCs and MS among the national population and analyze the urban–rural difference and whether insulin resistance plays a moderator effect in this association and (2) to describe the performance to predict MS using machine learning (ML) models.

**Design:**

A cross-sectional study was performed using 7,014 data from the China Health and Nutrition Survey (CHNS).

**Methods:**

WBCs were analyzed using an automatic hematology analyzer and MS was defined according to the criteria of the American Heart Association scientific statements of 2009. Variables on sociodemographic characteristics (sex, age, and residence), clinical laboratory (BMI and HOMA-IR), and lifestyle characteristics (smoking and drinking status) were used to construct ML models to predict MS [logistic regression (LR) and multilayer perceptron (MLP) neural network].

**Results:**

We found that 21.1% of participants (1,479/7,014) were classified as having MS. In multivariate logistic regression (including insulin resistance), the result revealed a significant positive association between WBCs and MS. The odds ratios (95% CI) for MS with increasing WBC level were 1.00 (reference), 1.65 (1.18, 2.31), and 2.18 (1.36, 3.50) (*p* for trend: 0.001). For two ML algorithms, two models showed adequate calibration and good discrimination, but the MLP showed better performance (AUC-ROC = 0.862 and 0.867).

**Conclusion:**

With the aim of confirming the association between WBCs and MS, this cross-sectional study is the first to show that maintaining normal WBC count levels is helpful to prevent the development of MS, and this association is independent of insulin resistance. The results also showed that the MPL algorithm represented a more prominent predictive performance to predict MS.

## Introduction

Metabolic syndrome (MS) is a pro-inflammatory disease characterized by a constellation of cardiovascular risk factors, including central obesity, elevated blood pressure (BP), dyslipidemia, and impaired glucose tolerance (1). With socio-economic development and lifestyle changes, the prevalence of MS has shown an increasing trend worldwide. Currently, approximately 25% of the global population suffers from MS (2). Therefore, exploring convenient, effective, and available methods for determining systemic inflammation statuses and diagnosing MS is urgently needed.

Numerous inflammatory markers have been widely used to assess systemic inflammatory statuses in clinical practice and public health practices. These markers included soluble adhesion molecules (such as E-selectin, P-selectin, intracellular adhesion molecule-1, and vascular cell adhesion molecule-1), cytokines (such as interleukin-1*β*, -6, -8, and -10, and tumor necrosis factor-*α*), acute-phase reactants [such as fibrinogen, serum amyloid A protein, and high sensitive C-reactive protein (hsCRP)], and white blood cell (WBC) count (3). Among these inflammatory markers, peripheral WBC counts are assayed in routine blood routine examination and used to diagnose systemic infection, tissue damage, and other conditions associated with inflammation (4). Furthermore, prior cross-sectional and cohort studies have shown that peripheral WBC count is associated with MS risk over the past decade (4–7). However, there are conflicting findings in recent studies. For example, two (8, 9) of the three studies (8–10) confirmed the cross-sectional and prospective findings. The association between WBC counts and MS may be different as there are urban–rural differences that have been observed in inflammation (11) and prevalence of MS (2). Additionally, insulin resistance plays an important pathophysiological role in the development of MS (12); however, it is unclear whether insulin resistance plays a moderator effect in this association (7, 13).

The recent progression of computer science has led to the development of machine learning (ML), a powerful tool that has been widely used to identify the risk factors of outcome variables. ML has provided the ability to compare the accuracy of the ML approaches and the traditional logistic regression in predicting the outcome variables (14). Therefore, in this study, we used a large-scale representative sample to comprehensively examine the association between WBC count and MS risk among the general Chinese population, and analyzed the urban–rural difference and whether insulin resistance plays a moderator effect in this association.

## Methods

### Study population

The China Health and Nutrition Survey (CHNS), established in 1989, is an ongoing prospective cohort study. A previous study has published a detailed study design (15). In brief, the multi-stage random cluster sampling method was used to select 4,400 households and 19,000 participants, which covers nine provinces (Guizhou, Guangxi, Heilongjiang, Henan, Hubei, Hunan, Liaoning, Jiangsu, and Shandong). So far, the CHNS has completed data collection from 11 waves (1989, 1991, 1993, 1997, 2000, 2004, 2006, 2009, 2011, 2015, and 2018). The CHNS has obtained clinical, dietary, anthropometric, and all other individual data from each household member. Because the collection and analysis of blood samples were only conducted and released in 2009, we therefore used data from the 2009 wave of the CHNS. A detailed study design has been described in a previous study (16).

We analyzed 12,178 individuals who agreed to participate and gave informed consent for analysis of their data. This study included participants aged ≥18 years who participated in the 2009 survey wave. Participants were excluded if they met any of the following criteria: (1) a history of diabetes (*n* = 131), hypertension (*n* = 1094), apoplexy (*n* = 38), or myocardial infarction (*n* = 28); and (2) incomplete data (*n* = 1963). After excluding these incomplete data, this study finally included 7,014 individuals (3,253 men and 3,761 women). In order to show that the sample size of this study has sufficient statistical power, sample size was calculated based on a previous study that determined the prevalence rate of MS [control group = 0.14 (514/3,556); experimental group = 0.39 (194/495)] in a Chinese population-based study (7). Statistical power for a two-sample proportion chi-squared test indicated that a total of 156 participants were included in this study, which could achieve 95% statistical power. Ethics approval was obtained from the Institutional Review Board of the University of North Carolina at Chapel Hill and the National Institute for Nutrition and Health, Chinese Center for Disease Control and Prevention.

### Blood biochemical measurement

Fasting blood samples were collected in blood collection tubes and tested in a national central lab in Beijing. Fasting blood glucose (FBG) and lipids [triglyceride (TG) and high-density lipoprotein cholesterol (HDL-C)] were analyzed using an automatic biochemical analyzer (Hitachi 7600 automated analyzer, Tokyo, Japan). Insulin was measured by an ELISA Kit (Millipore Corporation, Billerica, MA, USA). The homeostasis model assessment for insulin resistance (HOMA-IR) score was calculated using the following formula: fasting insulin (mU/L) * fasting glucose (mmol/L)/22.5 (17).

In order to determine all the above biochemical markers, FBG was measured by the glucose oxidase method. TG and HDL-C were measured by the enzymatic method.

### Assessment of WBC count

Fasting blood samples were drawn from the cubital vein for routine blood detection. WBC count was analyzed using an automatic hematology analyzer (Beckman Coulter LH751, Beckman Coulter, USA). Generally, an individual who maintains a very low leukocyte count level is healthier. We grouped the subjects into three categories—low level (subnormal level: ≦3.9), middle level (normal level: 3.91–9.94), and high level (above normal level: ≧10.0). We aimed to examine the association between a subnormal WBC count and risk of MS.

### Assessment of covariates

Anthropometric measurements (height, body weight, and waist circumference) were made using standard protocols. Body mass index (BMI) was calculated as the ratio of weight (kg) and height (m) squared. BP was measured three times after participants quietly sat for at least 5 min, and the maximum values of the first two of three readings were considered as the final BP values. All participants were also asked to fill in a standard questionnaire, which included demographic variables [sex, age, residence (city, suburban, town or county, capital city, and rural village)] and lifestyle variables [smoking status (smoker or not) and drinking status (drinker or not)].

### Definition of MS

According to the criteria of the American Heart Association scientific statements of 2009 (1), if a participant suffered from three or more of the following syndromes, he/she will be defined as having MS:

(1) Central obesity (≥90 cm in men, ≥80 cm in women);
(2) Elevated triglycerides (TG ≥1.7 mmol/L);
(3) Reduced HDL cholesterol (<1.0 mmol/L in men, <1.3 mmol/L in women);
(4) Elevated BP [systolic BP (SBP) ≥ 130 mmHg or diastolic BP (DBP) ≥ 85 mmHg]; and
(5) Elevated fasting glucose (≥5.6 mmol/L).

### Statistical analysis

All continuous variables were expressed as median [interquartile range (IQR)] because of non-normal distribution, and categorical variables were expressed as percentage. To examine differences in participants’ characteristics between WBC count category, Kruskal–Wallis test and chi-square test were applied for continuous variables and categorical variables, respectively. We examine the hypothesis of logistic regression analysis prior to its use. Firstly, to examine the independence of observation variables, interactions were tested by adding the respective multiplicative terms in the models simultaneously. There were significant interactions of WBC with age, residence, apoplexy, and insulin resistance on MS (*p* for interaction: <0.001, 0.038, <0.001, and 0.015, respectively) (**Appendix Table 1**). Based on the above findings, we also carried out subgroup analysis based on sex, age (age <65 years, age ≥65 years), residence (city, suburb, town, and village), apoplexy (yes or no), and insulin resistance (yes or no). Secondly, variance inflation factors (VIFs) were used to examine multicollinearity among explanatory variables, and the result shows that the VIFs of explanatory variables range from 1.02 to 1.91, which means that there is no multicollinearity between explanatory variables (**Appendix Table 2**). Thirdly, Cook’s distance was used to identify extreme outliers, and the results showed that Cook’s distance of each variable is less than or equal to 0.06, showing that no extreme outliers were found. Fourth, to initially assess whether there are linear relationships between continuous independent variables and dependent variable logit conversion values, the Box–Tidwell method was used (transformation of the independent variables). The results of the line test found nonlinear relationships between all continuous independent variables (age, BMI, and insulin resistance) and the dependent variable logit conversion values. To linearize relationships, all continuous variables were converted into ordinal categorical variables. To examine the association between WBC count and MS, multivariate logistic regression analyses were applied to calculate risk of MS in different WBC count categories when several potential confounding factors were adjusted. We adjusted for sex, age (continuous variable), BMI (continuous variable), residence (categorical variable), smoking and drinking status (categorical variable), and HOMA-IR.

To examine the robustness of the above-mentioned results, a sensitivity analyses was further conducted as follows: we excluded participants who was excessively thin and obese [BMI (<18 or >40 kg/m^2^)]. We also used receiver operating characteristic (ROC) curve analysis, precision, specificity, and sensitivity through ML including multilayer perceptron (MLP) and logistic regression (LR) to compare the predictive performance of the MS risk model. In all two-sided tests, *p*-values of <0.05 were defined as statistically significant. Stata 16.0 software (Stata Corp LP, College Station, TX, USA) was used for all tests.

## Results

### Participants’ characteristics according to WBC category

Participants’ characteristics according to WBC category are shown in **Table 1**; 3.4% of participants have a higher WBC level. Participants with a higher WBC level were more likely to be male, to be younger, and to live in a rural village. In addition, BMI and HOMA-IR increased statistically across different WBC categories. The proportion of participants with above-normal WBC count, who were cigarette smokers and alcohol drinkers, was statistically higher. However, there was no significant difference for other participants’ characteristics between WBC categories.

**Table 1:** Participants’ characteristics according to WBC counts category 1.

N= 7014	Low level (≦3.9)	middle level (3.91-9.94)	high level (≧10.0)	P value ^2^
N	397	6380	237	—
Sex, (males,%)	28.5	47.2	54.0	<0.001
Age, years	52.0 (40.0, 59.0)	47.0 (38.0, 58.0)	45.0 (33.0, 57.0)	0.001
Residence, %				
City	11.3	12.8	8.0	0.069
Suburb	15.9	18.2	15.2	0.269
Town	17.1	16.1	13.1	0.385
Rural village	55.7	52.9	63.7	0.003
BMI, Kg/m^2^	21.9 (20.0, 24.1)	23.1 (21.0, 25.6)	23.7 (21.3, 26.3)	<0.001
Cigarette smoker, %	14.6	31.3	40.1	<0.001
Alcohol drinker, %	22.4	34.4	35.9	<0.001
HOMA-IR	2.0 (1.4, 3.0)	2.3 (1.6, 3.4)	2.4 (1.6, 4.7)	<0.001

BMI = body mass index; HOMA-IR = homeostasis model assessment of insulin resistance.

1 Continuous variables are expressed as medians (interquartile range) and categorical variables are expressed as percentages.

2 P value were assessed using Kruskal Wallis test for continuous variables and chi-square test for categorical variables.

### Association of WBC category with MS and its components

**Figure 1:**
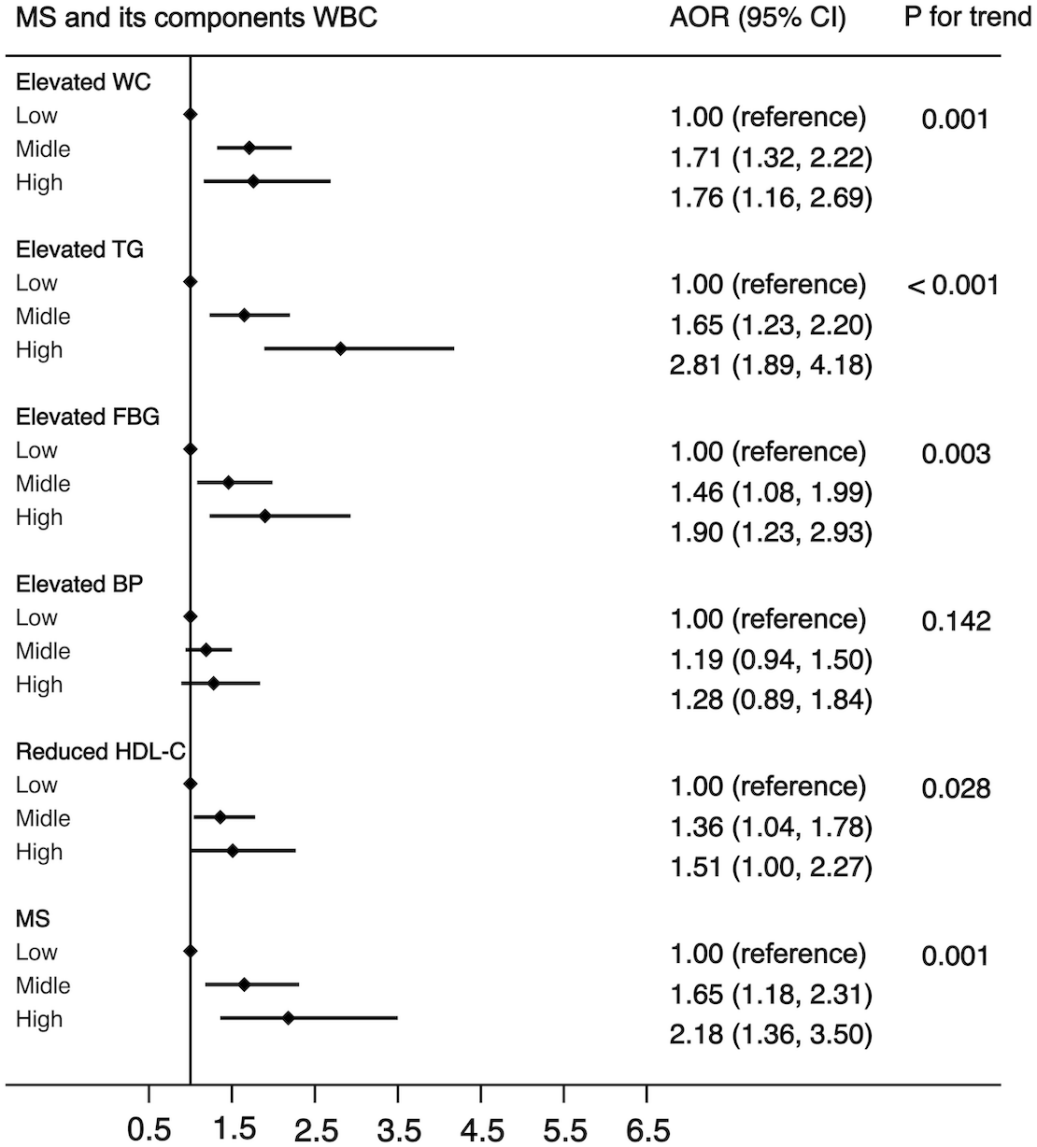
The association between WBC and risk of MS in all population. Adjusted for sex, age (<=39, 40-64, >=65), BMI (<25, >=25 & <30,>=30), residence (city, suburb, town or rural village), smoking (smoker or non-smoker) and drinking status (drinker or non- drinker), HOMA-IR (yes or no). Data are shown as OR and 95% CI.

**Figure 2:**
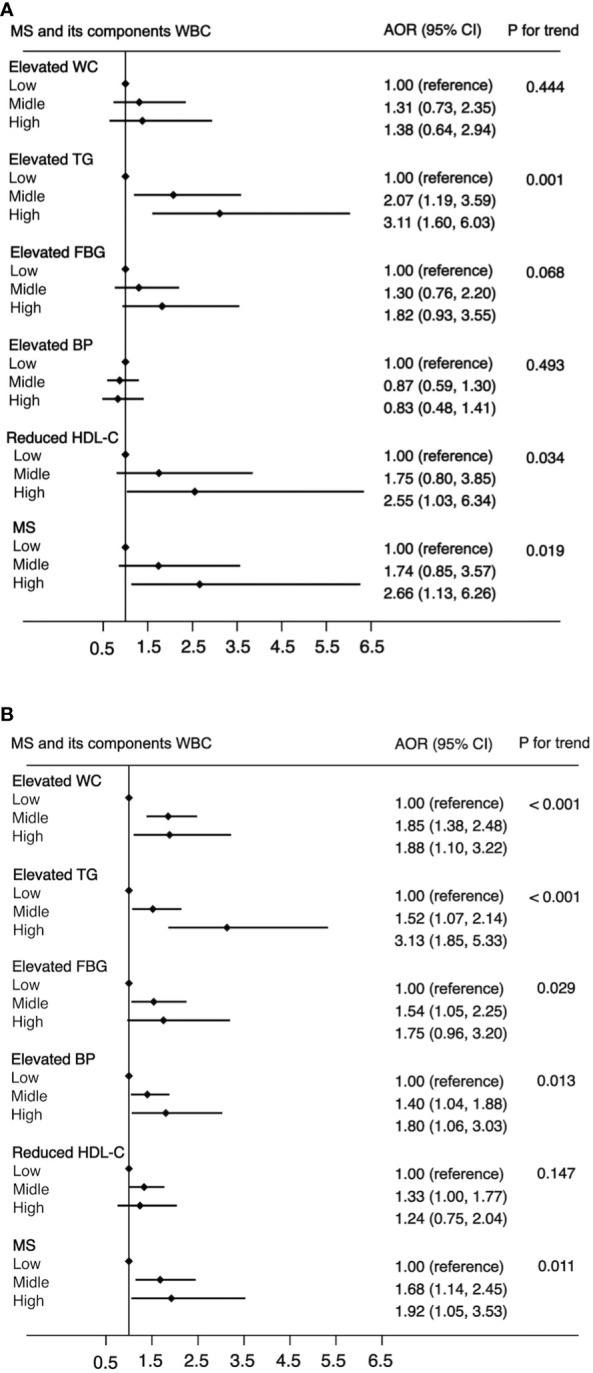
The association between WBC and risk of MS in male **(A)** and female **(B)**. Adjusted for age (<=39, 40-64, >=65), BMI (<25, >=25 & <30,>=30), residence (city, suburb, town or rural village), smoking (smoker or non-smoker) and drinking status (drinker or non-drinker), HOMA-IR (yes or no). Data are shown as OR and 95% CI.

In all 7,014 participants, 1,479 were diagnosed with MS. The prevalence rates of MS in the low level, middle level, and high level were 12.1% (48 of 397), 21.4% (1,368 of 6,380), and 26.6% (63 of 237), respectively. **Figure 1** shows a positive association between WBC count and risk of MS and its components. After adjustment for a potential confounder, the adjusted ORs for MS across WBC category were 1.00 (reference) for low level, 1.65 (95%CI: 1.18, 2.31) for middle level, and 2.18 (95%CI: 1.36, 3.50) for high level (*p* for trend, 0.001). Similar significant relationships were also found when components of MS were analyzed respectively (**Figure 1**). In addition, a higher WBC level was also associated with increased risk of MS in both male and female (**Figure 2**).

### Subgroup analyses stratified by sex, age, residence, smoking status, drinking status, BMI, and insulin resistance

**Figure 3:**
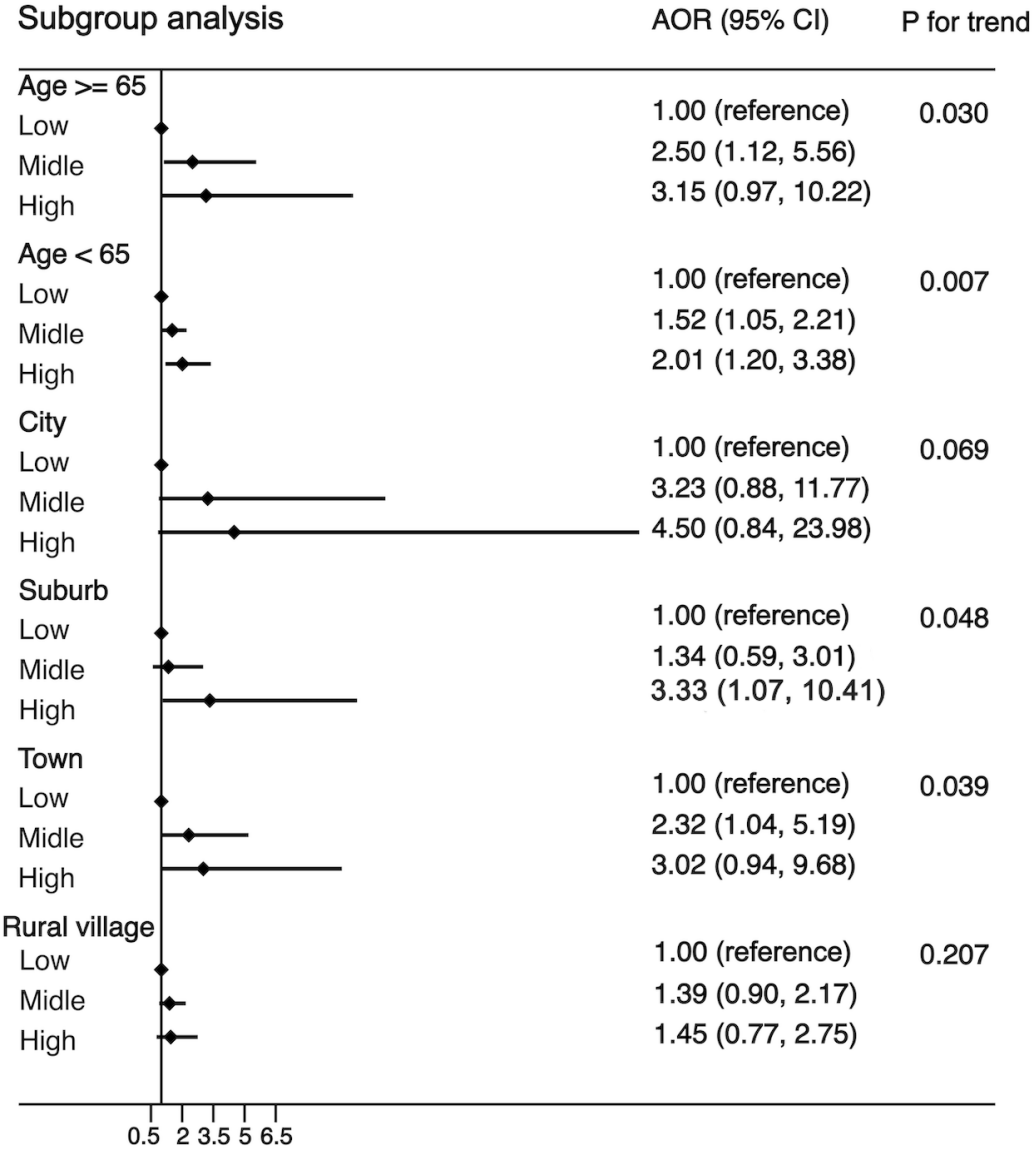
Stratification analysis of the association between WBC and risk of MS. Subgroups based on age (age <65 years, age ≥65 years) or residence (city, suburb, town or rural village). Data are shown as OR and 95% CI.

**Figure 4:**
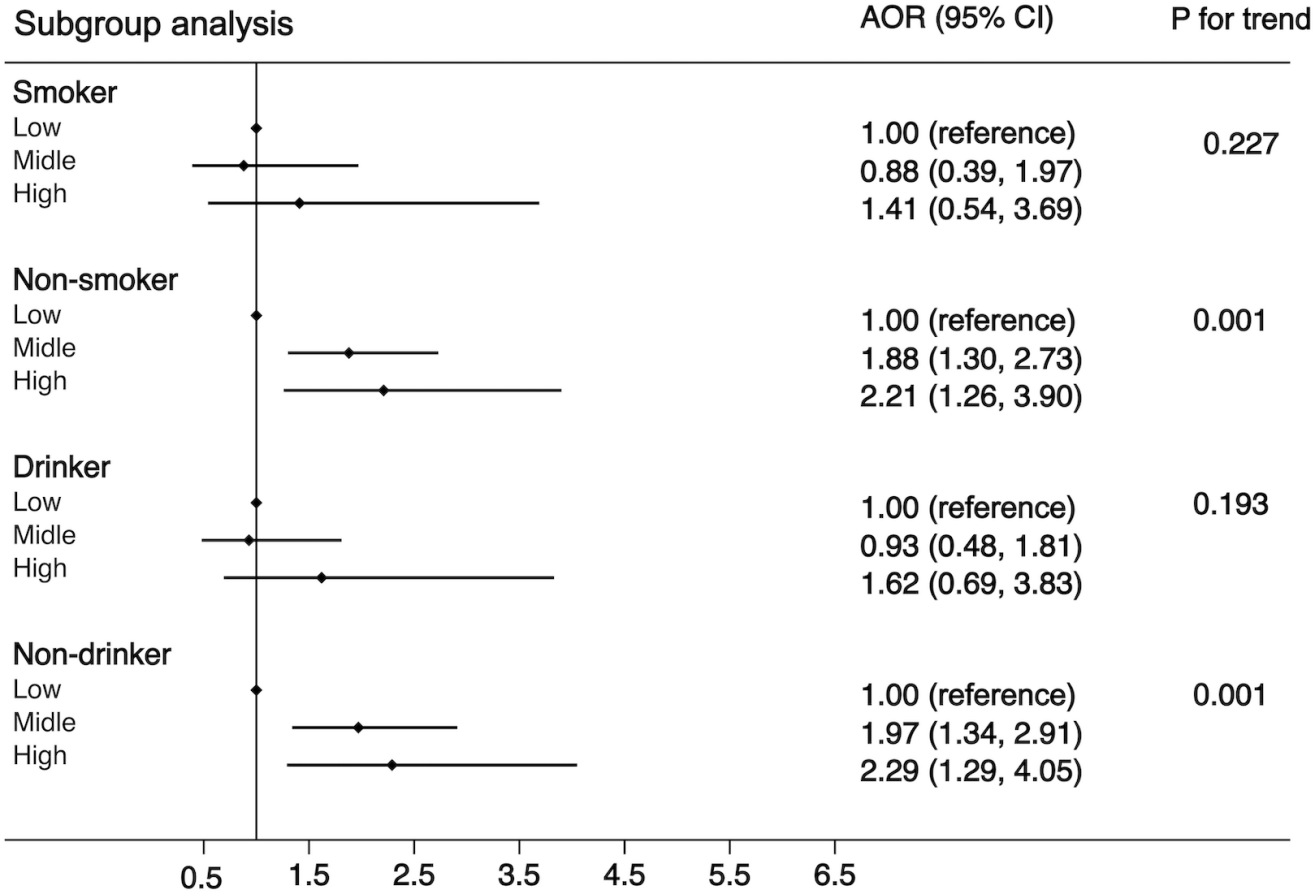
Stratification analysis of the association between WBC and risk of MS. Subgroups based on smoking (smoker or non-smoker) or drinking status (drinker or non-drinker). Data are shown as OR and 95% CI.

**Figure 5:**
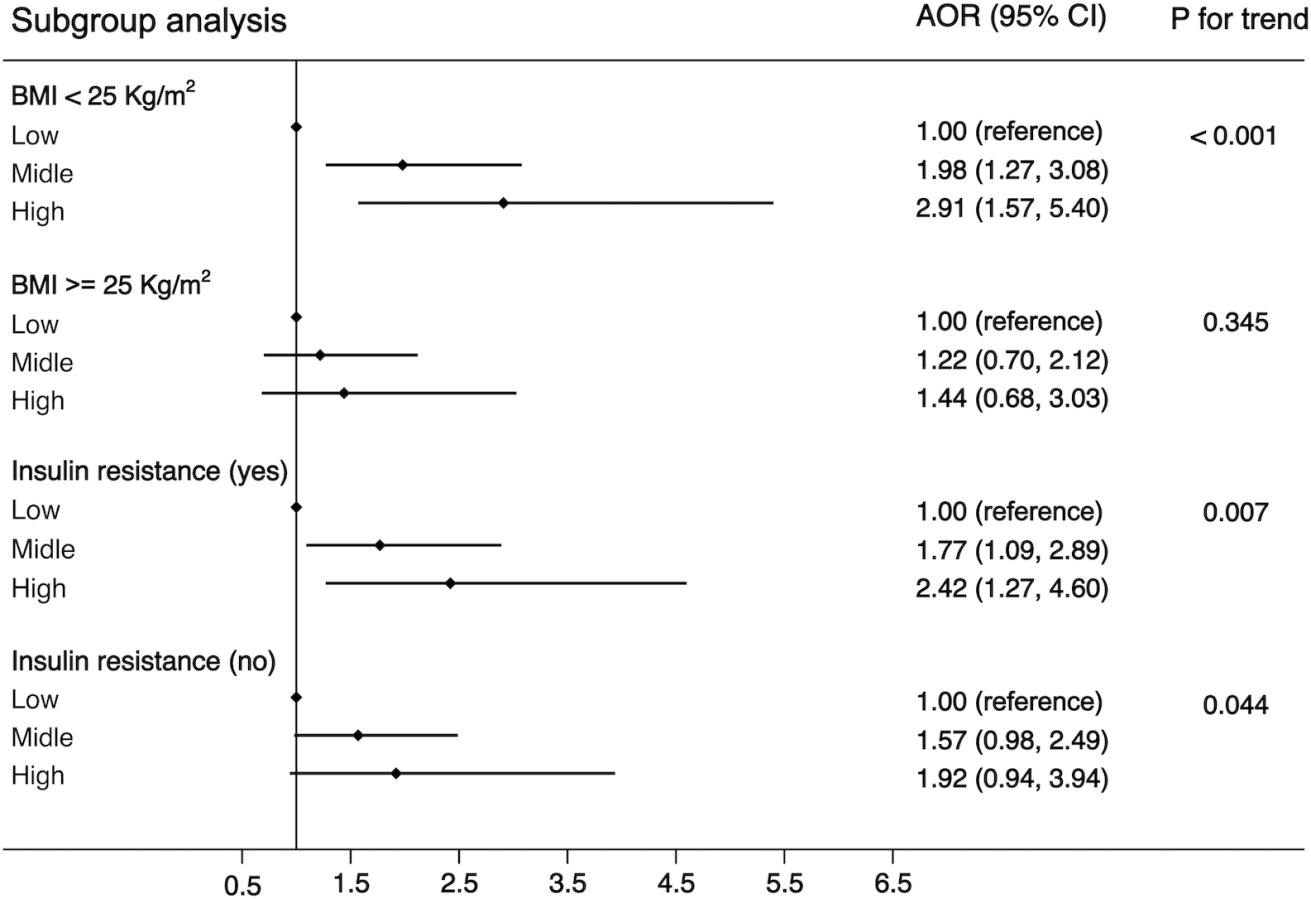
Stratification analysis of the association between WBC and risk of MS. Subgroups based on BMI (<25 or >=25) or HOMA-IR (yes or no). Data are shown as OR and 95% CI.

Another purpose of this study was to examine the associations of WBC category and MS by the following subgroups: age (<65 or ≧65 years), residence (city, suburban, town, or rural village), smoking status (smoker or non-smoker), drinking status (drinker or non-drinker), BMI (<25 kg/m^2^ or ≧25 kg/m^2^), and insulin resistance (yes or no). The strength of the association between WBC count and MS increased with age (OR, 1.42 [95%CI, 1.10-1.83] for adults aged <65 years; 1.85 [95%CI, 1.06, 3.21] for those aged ≧65 years). The strength of the association between WBC count and MS was weaker in participants who resided in a rural village (OR, 1.22 [95% CI, 0.90–1.66]) compared with other residences. The association between WBC count and MS was stronger in non-smokers (OR, 1.55 [95% CI, 1.19–2.03]) and non-drinkers (OR, 1.56 [95% CI, 1.19–2.04]) compared with smokers (OR, 1.34 [95%CI, 0.83-2.14))/drinkers (OR, 1.35 [95% CI, 0.86–2.12]). The association between WBC count and MS was stronger in normal/underweight participants compared with overweight/obese participants (OR, 1.71 [95% CI, 1.27–2.30] *vs*. 1.20 [95% CI, 0.82–1.74]) and was equivalent whether there was insulin resistance or not (OR, 1.54 [95% CI, 1.12–2.10] *vs*. 1.42 [95% CI, 1.01–1.99]). The detailed results has been showed in **Figures 3**–**5**. Distribution of components of MS according to WBC category is also shown in violin plots (**Figure S1**).

### Sensitivity analyses

The aforementioned findings remained stable in a sensitivity analysis (**Table S1**). The results remained significant with the exclusion of excessively thin and obese participants [BMI (<18 or >40 kg/m^2^)].

To assess the predictive performance of predictive models, we selected MLP and logistic regression to compare their accuracy. The AUROC was calculated both in the 70% of training set and in the 30% of test set. It is generally accepted that an AUROC curve greater than 0.5 indicated having an adequate predictive performance. The results showed that the MPL algorithm represented a more prominent predictive performance (AUROC in the training set: 0.814; AUROC in the testing set: 0.842) compared to other algorithms (**Table S2**).

## Discussion

In this study, we comprehensively examined the association between WBC count and the risk of MS in a representative Chinese population, and the results revealed that participants with a higher WBC count had a higher risk of MS and its components than those with a lower WBC count after adjusting for potential confounders. This study obtained the following novel results: (1) the significant associations between WBC count and risk of MS occur in participants living in suburbs or cities but not in towns or rural villages; (2) these significant associations were independent of insulin resistance; and (3) the two models showed adequate calibration and good discrimination, but the MLP showed better performance.

The current cross-sectional study is consistent with the results of previous cross-sectional and cohort studies (4–7), which suggested that a higher WBC count level was related to an increased risk of MS. However, the association with WBC count varied between components of MS. Overall, the previous cross-sectional and prospective studies were heterogeneous, with differences in WBC count category, confounding factor adjustments, and sample selections. Nevertheless, the robust significant positive association between WBC count and MS was retained, despite the challenges introduced by these heterogeneities. This study harmonized fasting blood sample and other data across nine provinces, which largely attenuated these heterogeneities. Insulin resistance is an important marker for MS and its components; therefore, the significant positive associations of WBC count with the risk of MS and its components remained unchanged after adjusting for insulin resistance.

There are several plausible mechanisms that explain how WBC count may increase MS risk. Firstly, it is well known that insulin resistance is considered to be the root cause of MS (12); therefore, insulin resistance may mediate the association between WBC count and MS. Pro-inflammatory cytokines may lead to the activation of protein kinases and subsequently negatively regulate insulin receptor substrate and reduce the expression of glucose transporter 4 (18, 19); this inhibits the efficiency of blood glucose uptake (20). Therefore, compensatory hypersecretion of insulin can induce insulin resistance. However, the results of the current study suggest that elevated WBC count may increase the risk of MS in Chinese adults, even after adjusting for insulin resistance.

Secondly, it has been suggested that pro-inflammatory cytokines can increase hepatic fatty acid synthesis and stimulate lipolysis, which promotes the movement of free fatty acids to the liver (21). The above two mechanisms will subsequently enhance the production and secretion of triglycerides in the liver, resulting in hypertriglyceridemia (21).

Thirdly, the observed association between WBC count and elevated BP may be related to impaired endothelial function. Inflammation causes vasodilation, and the antithrombotic and antiatherosclerotic functions of the vascular endothelium are prevented by limiting the production of nitric oxide and prostacyclin (22). In contrast, the WBCs bind to the vascular endothelium, which may cause an increase in leukocytosis in capillaries, subsequently inducing capillary stenosis and increasing vascular pressure (23, 24), which may eventually lead to elevated BP.

Fourth, accumulated visceral fat can induce elevated fasting glucose levels compared to subcutaneous fat. This is because excessive free fatty acids produced by visceral fat through catabolism will overflow from adipose tissue into islet cells and induce fat ectopic deposition, causing insulin resistance in the muscle, liver, and pancreatic β cells, which subsequently can result in impaired glucose uptake (25). An imbalance between glucose production and uptake generally leads to elevated fasting glucose levels.

Logistic regression is a linear model widely used to analyze the linear association between independent and dependent variables (dichotomous variables), while ignoring the complex relationship between independent variables. MPL is composed of an input layer, one or more hidden layers, and an output layer, and can analyze the complex and potential associations among variables, rather than being limited to the linear relationship between the input and output (26). The results of this study indicated that the MPL algorithm had superior performance in predicting MS compared to logistic regression, indicating that the MPL algorithm is more suitable for predicting MS.

This study has several limitations. Firstly, the cross-sectional design of this study made it difficult to confirm the causal relationship between WBC count and MS and its components. Secondly, because there are fewer participants with a higher WBC level, this study therefore found seemingly inflated OR in **Figures 2**, **3**, indicating that there are some sparse effects that may increase the probability of monotonic likelihood (27). Thirdly, the cross-sectional studies had Neyman bias because we could not identify whether the participants had suffered from MS in the past or only in the present. Therefore, the results of this study may be affected by prevalence–incidence (Neyman’s) bias (28, 29). Further prospective cohort studies, which select the newly identified cases as study participants, will effectively avoid Neyman bias.

## Conclusion

This study reveals representative evidence of positive associations between WBC counts and MS as well as its components in a Chinese population. The findings of this study indicate that WBC count, as a convenient and routine examination, could be potentially used as a risk marker in the early identification and prevention of MS.

## Data availability statement

Publicly available datasets were analyzed in this study. This data can be found here: https://www.cpc.unc.edu/projects/china.

## Ethics statement

Ethics approval was obtained from the Institutional Review Board of the University of North Carolina at Chapel Hill and the National Institute for Nutrition and Health, Chinese Center for Disease Control and Prevention. The patients/participants provided their written informed consent to participate in this study.

## Author contributions

ZR and LL designed this study, performed the statistical analyses, and wrote the manuscript. ZR and LL revised the manuscript. ZR and SL provided constructive and editorial feedback on drafts of the manuscript. All authors contributed to the article and approved the submitted version.

## Supplementary material

Supplementary Figure 1 Violin plot of components of MS based on different WBC category among all participants.
