# Supplementary figures and images for: The positive association between white blood cell count and metabolic syndrome is independent of insulin resistance among a Chinese population: a cross-sectional study
Source: Front Immunol. 2023 Apr 28;14:1104180. doi: 10.3389/fimmu.2023.1104180 (PMC10175667; doi:10.3389/fimmu.2023.1104180)

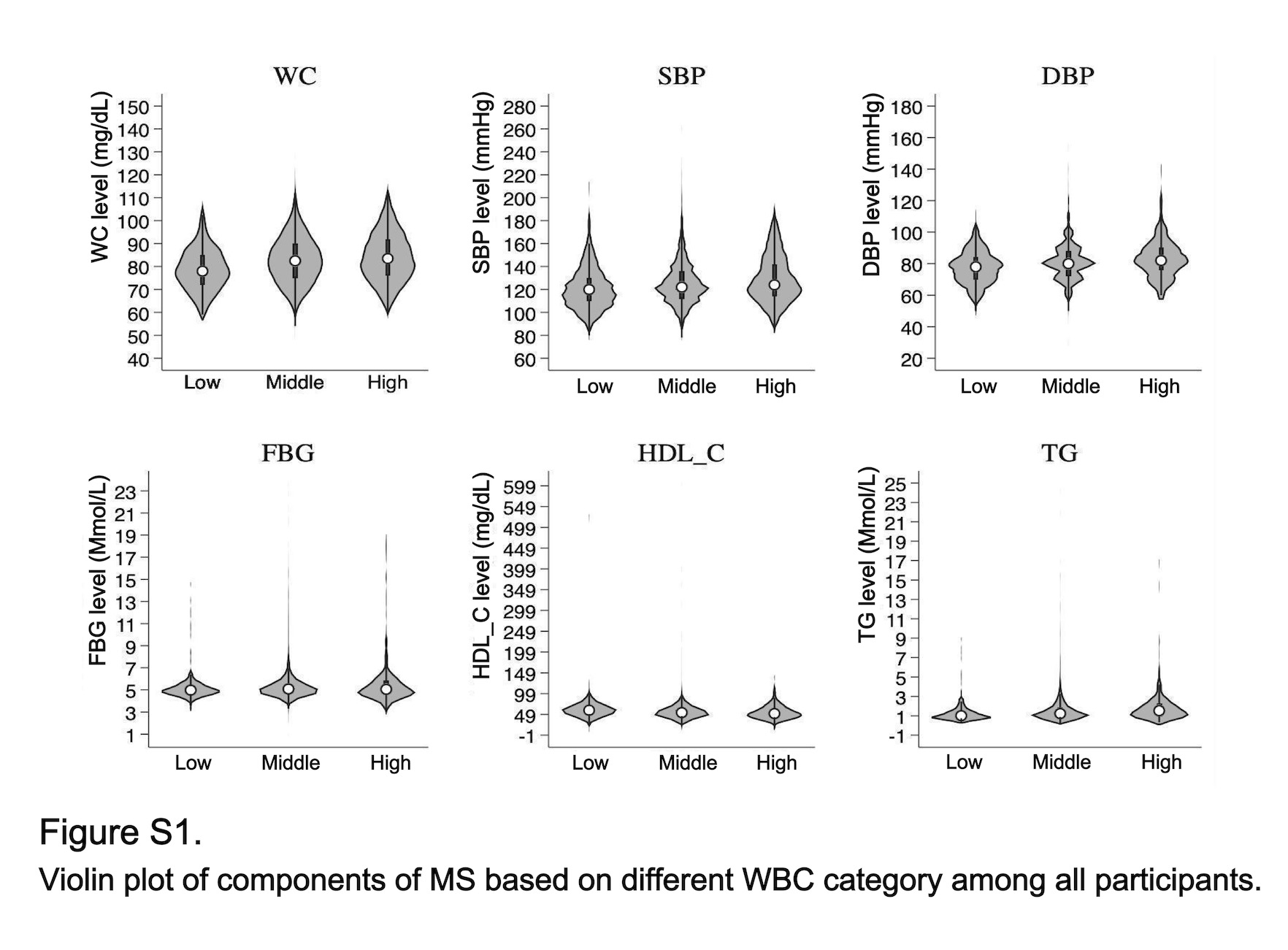

Supplement: Supplementary Figure 1 — Violin plot of components of MS based on different WBC category among all participants. [file Image_1.jpg]
