# Supplementary material for: The positive association between white blood cell count and metabolic syndrome is independent of insulin resistance among a Chinese population: a cross-sectional study
Source: Front Immunol. 2023 Apr 28;14:1104180. doi: 10.3389/fimmu.2023.1104180 (PMC10175667; doi:10.3389/fimmu.2023.1104180)
Supplement: Supplementary file 2 [file Table_1.docx]

**Table S1 Sensitivity analysis for the association between white cell counts category and risk of metabolic syndrome**

| n=6693 | Sensitivity analysis |
| --- | --- |
| WBC counts category | OR(95% CI) |
| Low level (subnormal level) | 1.00 (reference) |
| Middle level | 1.68 (1.19, 2.36) |
| High level (above normal level) | 2.20 (1.37, 3.55) |
| P for trend ^d^ | 0.001 |

Models were adjusted for sex (categorical variable), age (<=39, 40-64, >=65), BMI (<25, >=25 & <30, >=30), residence (categorical variable), smoking (categorical variable) and drinking status (categorical variable), insulin resistance (categorical variable). Sensitivity analysis : Excluding participants having excessively thin and obesity [BMI (< 18 or > 40 kg/m2)].

**Table S2 Predictive Performance Expressed in Area Under Receiver Operating Characteristic Curve, Sensitivity and Specificity, of the Three Models Considered to Predict Metabolic Syndrome.**

|  | N=7014 | AUROC | Precision | Accuracy | Specificity | Sensitivity |
| --- | --- | --- | --- | --- | --- | --- |
| Training set  （70%） | LR | 0.805 | 0.204 | 0.817 | 0.834 | 0.464 |
|  | MLP | 0.814 | 0.364 | 0.818 | 0.847 | 0.612 |
| Testing set  （30%） | LR | 0.814 | 0.300 | 0.815 | 0.839 | 0.604 |
|  | MLP | 0.842 | 0.399 | 0.829 | 0.858 | 0.637 |

LR: logistic regression; MLP: multilayer perception.

**Table S3 Negative Binomial regression for the association between WBC and risk of MS.**

| n=7014 | OR(95% CI) |
| --- | --- |
| WBC counts category |  |
| Low level (subnormal level) | 1.00 (reference) |
| Middle level | 1.26 (1.14, 1.39) |
| High level (above normal level) | 1.43 (1.25, 1.64) |
| P for trend ^d^ | <0.001 |

Models were adjusted for sex (categorical variable), age (<=39, 40-64, >=65), BMI (<25, >=25 & <30, >=30), residence (categorical variable), smoking (categorical variable) and drinking status (categorical variable), insulin resistance (categorical variable).
