# Supplementary material for: The positive association between white blood cell count and metabolic syndrome is independent of insulin resistance among a Chinese population: a cross-sectional study
Source: Front Immunol. 2023 Apr 28;14:1104180. doi: 10.3389/fimmu.2023.1104180 (PMC10175667; doi:10.3389/fimmu.2023.1104180)
Supplement: Supplementary file 3 [file Table_2.pdf]

Appendix Table 1. Significances for the interaction between WBC and potential covariates.

Multiplicative interaction terms	P for interaction
WBC*sex	0.647
WBC* age	**<0.001**
WBC* residence	**0.038**
WBC* BMI	0.714
WBC* HOMA-IR	**<0.001**
WBC* cigarette smoke	0.587
WBC* alcohol drink	0.756
WBC* History of disease (hypertension)	0.906
WBC* history of disease (diabetes)	0.900
WBC* history of disease (apoplexy)	**0.015**
WBC* history of disease (myocardial Infarction)	0.641

Appendix Table 2. Examination of multicollinearity among explanatory variables

Variable	VIF	1/VIF
Sex	1.910	0.524
Cigarette smoker	1.710	0.585
Alcohol drinker	1.500	0.668
History of disease (hypertension)	1.200	0.837
Age	1.130	0.889
History of disease (diabetes)	1.070	0.931
BMI	1.070	0.932
HOMA-IR	1.040	0.957
History of disease (diabetes)	1.040	0.958
History of disease (myocardial Infarction)	1.030	0.970

Residence	1.030	0.975
White cell counts	1.020	0.979
Mean VIF	1.230	

Appendix Table 3. Negative binomial regression analysis of WBC and risk of MS adjusting potential covariates.

N =3869	No. of participants/cases	Model 1 [a]	Model 2 [b]
**Metabolic syndrome**			
Category 1 (subnormal level)	13/125	1.00 (reference)	1.00 (reference)
Category 2	808/3581	1.51 (1.18, 1.94)	1.47 (1.15, 1.89)
Category 3 (above normal level)	45/155	1.90 (1.39, 2.60)	1.76 (1.28, 2.40)
**P for trend [d]**	—	<0.001	0.001
